# *Gasterophilus* in horses from Romania: diversity, prevalence, seasonal dynamics, and distribution

**DOI:** 10.1007/s00436-024-08419-3

**Published:** 2024-12-23

**Authors:** Ș. O. Rabei, A. S. Cârstolovean, C. A. Culda, A. D. Mihalca

**Affiliations:** Department of Parasitology and Parasitic Diseases, Faculty of Veterinary Medicine, University of Agricultural Sciences and Veterinary Medicine of Cluj-Napoca, Calea Manaștur 3-5, 400372 Cluj-Napoca, Romania

**Keywords:** Horses, *Gasterophilus*, Epidemiology, Romania, Abattoirs

## Abstract

This is the first study that targets the epidemiology of *Gasterophilus* spp. in slaughtered horses from Romania. Previously to our research, there were five recorded species: *Gasterophilus haemorrhoidalis*, *Gasterophilus inermis*, *Gasterophilus intestinalis*, *Gasterophilus nasalis*, and *Gasterophilus pecorum* with a dispersed distribution throughout the country, the data being recorded more than 73 years ago. The collection of *Gasterophilus* larvae was carried out from the digestive system (stomach, duodenum, and rectum) of horses in three abattoirs, monthly between January 2023 and December 2023. Following dissection, all larvae were stored in absolute ethanol until morphological and molecular identification. The larvae were counted and identified according to morphological keys. Overall, 394 horses were examined, of which 211 (53.6%) were infected with L2 or L3 of *Gasterophilus* spp. Only two species were found: *G. intestinalis* (prevalence 52.0%) and *G. nasalis* (14.0%). Coinfection with both species occurred in 49 horses (12.4%). In total, 9759 larvae were collected (7916 L3 and 1843 L2). Larvae were localized in the stomach (*G. intestinalis*) and duodenum (*G. nasalis*) of horses. There was a strong seasonal variation in the mean intensity and mean abundance for both *G. intestinalis* and *G. nasalis* larvae and also for coinfections (*p* < 0.05). There were no significant differences between prevalence and mean intensity across gender, group age, or horse colour. Our findings show that *Gasterophilus* infection is widespread within Romania territory, being a possible cause of illness and welfare issues in horses.

**Supplementary Information:**

The online version contains supplementary material available at 10.1007/s00436-024-08419-3.

## Background

*Gasterophilus* spp., with nine valid species, are highly host-specific parasites that cause myiasis in equids such as horses, donkeys, and zebras (Zumpt 1965; Colwell et al. 2006; Li et al. 2019). The pathology associated with *Gasterophilus* spp. larvae is related to the location in the host, the larval developmental stage, the species involved (Principato 1988), and the intensity of infestation. In general, the digestive tract lesions consist of ulcerations, mucosal atrophy, and fibrosis, but also peritonitis and occasional intestinal obstructions. Moreover, *Gasterophilus* can cause systemic effects such as anaemia, malabsorption, and even death when a high number of larvae are present (Cogley and Cogley 1999; Otranto et al. 2005; Sánchez-Andrade et al. 2010).

Although most infestations are asymptomatic, the disease can be linked to animal welfare issues, as equids can show stress signs in the presence of flies (Cogley and Cogley 1999), with increased levels of cortisol (Christensen et al. 2022). On rare occasions, *Gasterophilus* spp. can also affect non-equid hosts such as cattle (Cope and Catts 1991) or even humans (Anderson 2006).

The life cycle of *Gasterophilus* spp*.* lasts about 12 months, while the life span of each stage can vary between species. The ratio between the duration of larval stages and the total life cycle is approximately 5/6 (Li et al. 2018). The pupal period lasts between 12 and 34 days, while L1 is ready to hatch within 10 days after the deposition of the eggs. The longevity of adults varies between 1 and 26 days (Anderson 2006; Li et al. 2019). Aspects like pupal development rate, flying activity, egg deposition, or egg hatching are linked to minimum temperature thresholds, as well as humidity values (Sweeney 1990; Pilo et al. 2015; Zhang et al. 2021). Considering the adults’ short life span with the requirements for certain environmental factors and climate change, the epidemiology of *Gasterophilus* spp. may significantly vary from one generation to another. In opposition with the importance of the external factors, the intrinsic host-related factors such as age, sex, or colour have not been found to be significant for the level of *Gasterophilus* spp. infestation (Bucknell et al. 1995; Agneessens et al. 1998; Otranto et al. 2005; Pilo et al. 2015).

Although the genus *Gasterophilus* has a worldwide distribution, each species has its specific geographical pattern, with differences also in prevalence and intensity (Otranto et al. 2005; Rodrigues Felix et al. 2007; Rehbein et al. 2013). Out of the nine species, only *G. intestinalis* and *G. nasalis* have a worldwide distribution (Li et al. 2019). Historically, in Romania, five species have been recorded: *G. intestinalis*, *G. nasalis*, *G. haemorrhoidalis*, *G. inermis*, and *G. pecorum* (Dinulescu 1961). However, the data is rather old, and the current diversity and epidemiology have not been investigated in the last 73 years despite significant climate, land use, social, and horse demographic changes. In this context, the aim of our study consisted of mapping the distribution of *Gasterophilus* spp. in Romania and evaluating their current species diversity, prevalence, and monthly dynamics over a period of one year, using as indicator slaughtered horses.

## Materials and methods

### Horses and study area

**Fig. 1:**
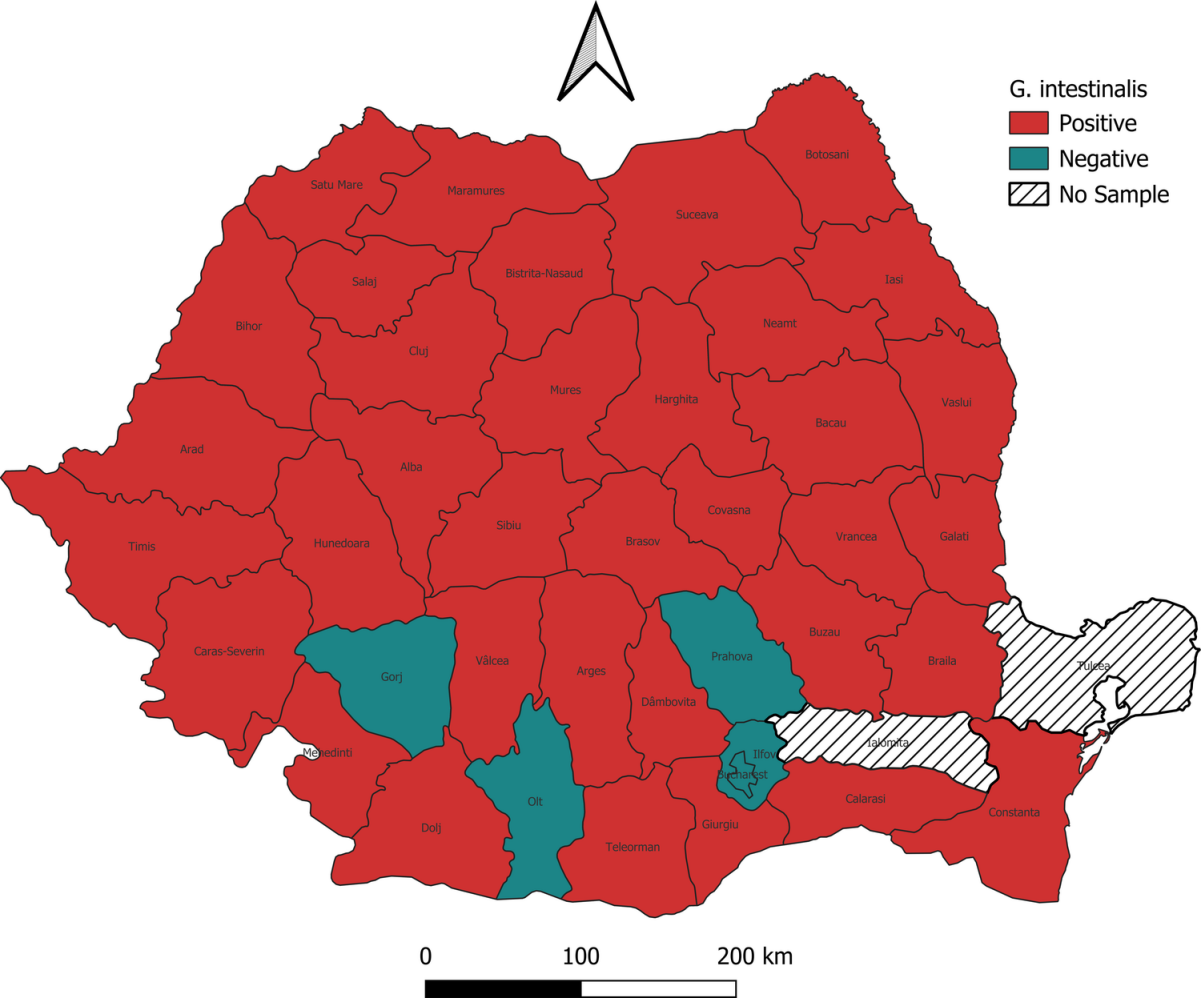
The distribution of *G. intestinalis* in Romania

**Fig. 2:**
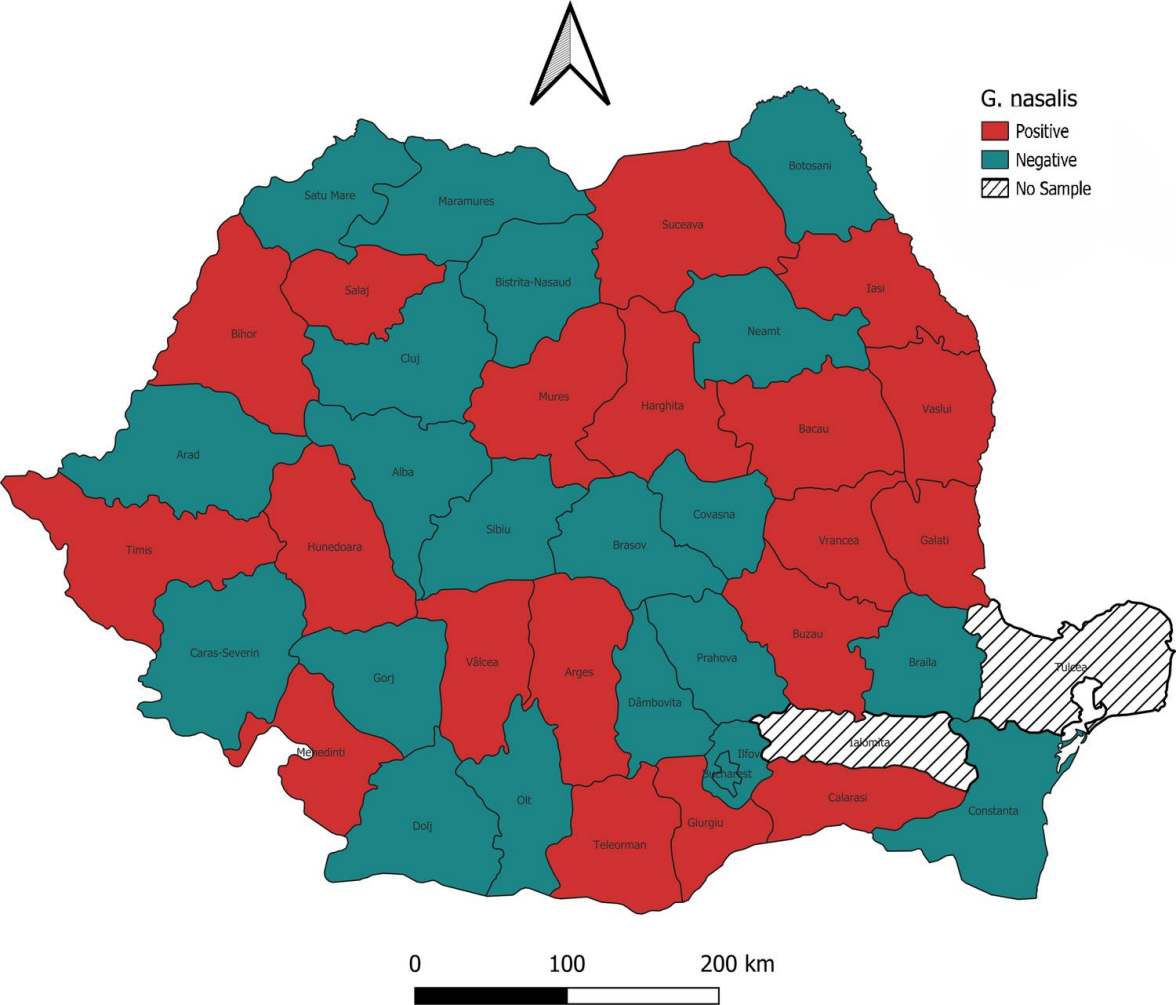
The distribution of *G. nasalis* in Romania

In order to achieve a sufficient number of samples that covers the majority of Romania territory, we selected abattoirs (Girișu de Criș—Bihor county, Nojag—Hunedoara county, Călinești—Suceava county), with a permanent activity of slaughtered horses, gathered from multiple counties across Romania. For each horse, the following data was recorded: sampling date, age, sex, colour, and origin at the county level (see supplementary file). As a result, we collected samples from a total of 394 slaughtered horses, with a distribution that covered 39 out of 41 counties (Figs. 1 and 2). Overall, the gathered horse population can be characterized by a comparable number of genders (216 females and 176 males), with an age that ranged between 5 and 472 months, and a total of 12 colour variants.

### Sampling

The digestive systems (stomach, duodenum, rectum) of 394 horses were collected monthly between January and December 2023. The digestive tubes were carefully dissected, and all larval stages of *Gasterophilus* were collected, washed in NaCl 0.9%, and preserved in absolute ethanol until identification, grouped in tubes based on their location in the host.

### Morphological species identification of *Gasterophilus* larvae

The morphological characteristics of each larva were observed under an Olympus SZX16 stereo-zoom microscope. Pictures and focus stacking were done with an Olympus SC180 camera and dedicated software. The larvae were identified according to morphological keys (Li et al. 2018; Zumpt 1965).

### Molecular species identification of *Gasterophilus* larvae

The molecular analysis was performed on 26 randomly selected larvae (18 *G. intestinalis* and 8 *G. nasalis*), using a section of the abdominal segments. The genomic DNA was extracted using the DNeasy Blood and Tissue (Qiagen, Dusseldorf, Germany) following the manufacturer instructions. The DNA was amplified by targeting an approximately 650-bp region of the mitochondrial COI region using the following primers: LCO1490-L (5′-GGTCWACWAATCATAAAGATATTGG-3′) and HCO2198-L (5′-RAAACTTCWGGRTGWCCAAARAATCA-3′) (Folmer et al. 1994). PCR reaction and amplification conditions were conducted according to Horváth et al. (2021) using a C1000™ Thermal Cycler (Bio-Rad, London, UK). The positive amplicons obtained were purified using the Gel/PCR DNA Fragments Extraction Kit (Geneaid) and sent to Macrogen Europe in Amsterdam, Netherlands, for bidirectional sequencing. The sequences were assembled in Geneious Prime (Biomatters Ltd., Auckland, New Zealand) (Kearse et al. 2012) and compared with those listed in GenBank™ applying the BLAST algorithm.

### Statistical analysis and mapping

The statistical analysis was performed in order to establish the possible correlations between categorical data such as month and season of sampling and host gender, colour, and age, on one side, and the data on distribution, intensity (i.e. number of larvae) (total = L3 + L2, L3, and L2), mean intensity, and mean abundance. For further statistical analysis, the horses were grouped into three age groups (< 3 years, 3–10 years, > 10 years) and three colour categories (1—white, grey, spotted black; 2—bay, brown, buckskin, chestnut, palomino, roan, and strawberry roan; 3—black, smoky black). The statistical significance between distribution and host data was assessed by performing the chi-square (*χ*^2^) test, while the relationship between larval intensity and host data was analysed by the Kruskal–Wallis test. Following the Kruskal–Wallis test results, post hoc multiple comparisons were performed using Dunn’s method with a Bonferroni correction. The statistical methods mentioned above were performed using IBM S.P.S.S. 20.0 software. Maps were created through QGis version 3.34.2 software (2024; https://www.qgis.org/en/site).

## Results

**Fig. 3:**
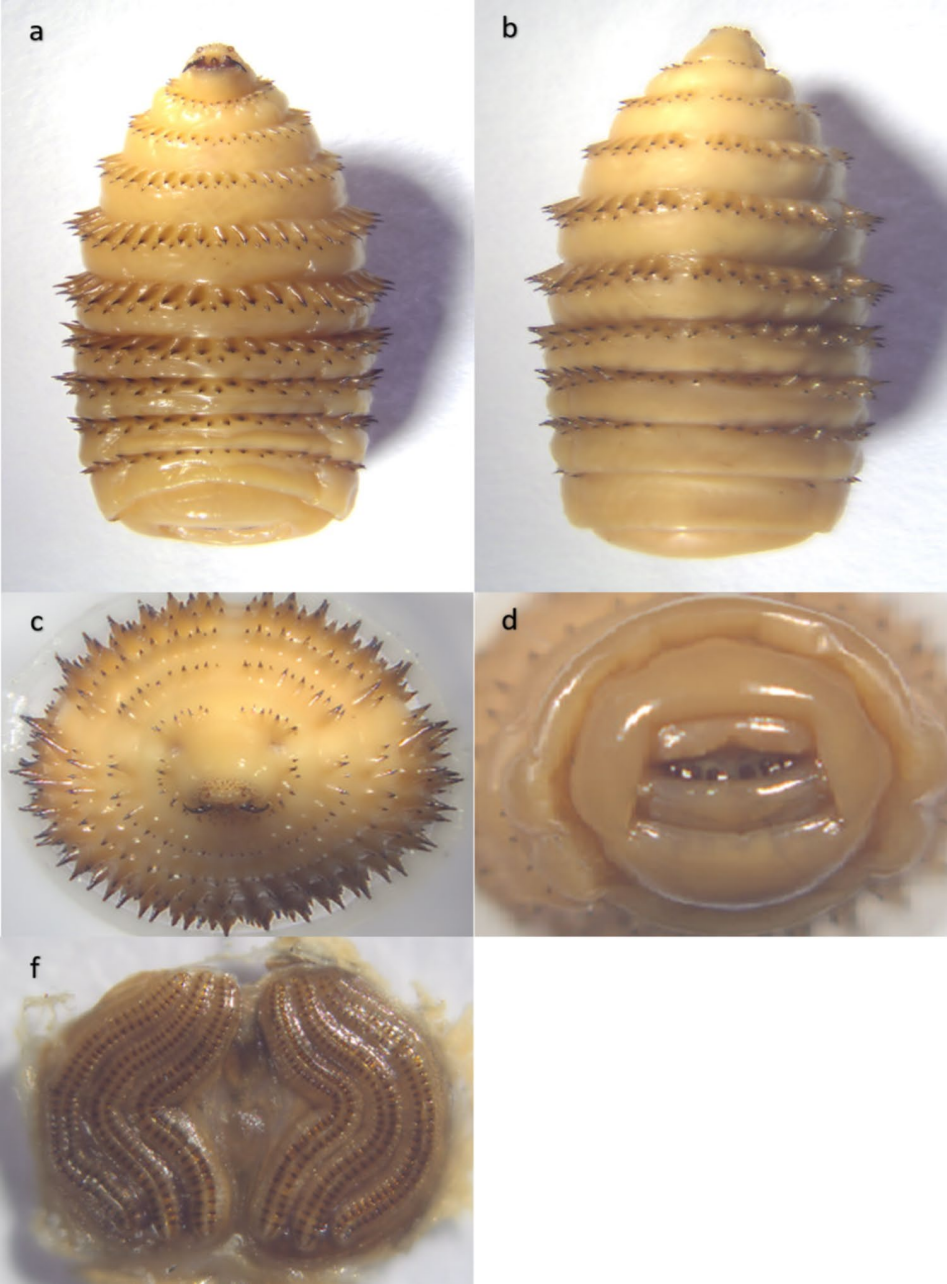
*G. intestinalis* L3 morphology from **a** ventral, **b** dorsal, **c** cranial, **d** caudal, and **e** spiracular plates view

**Fig. 4:**
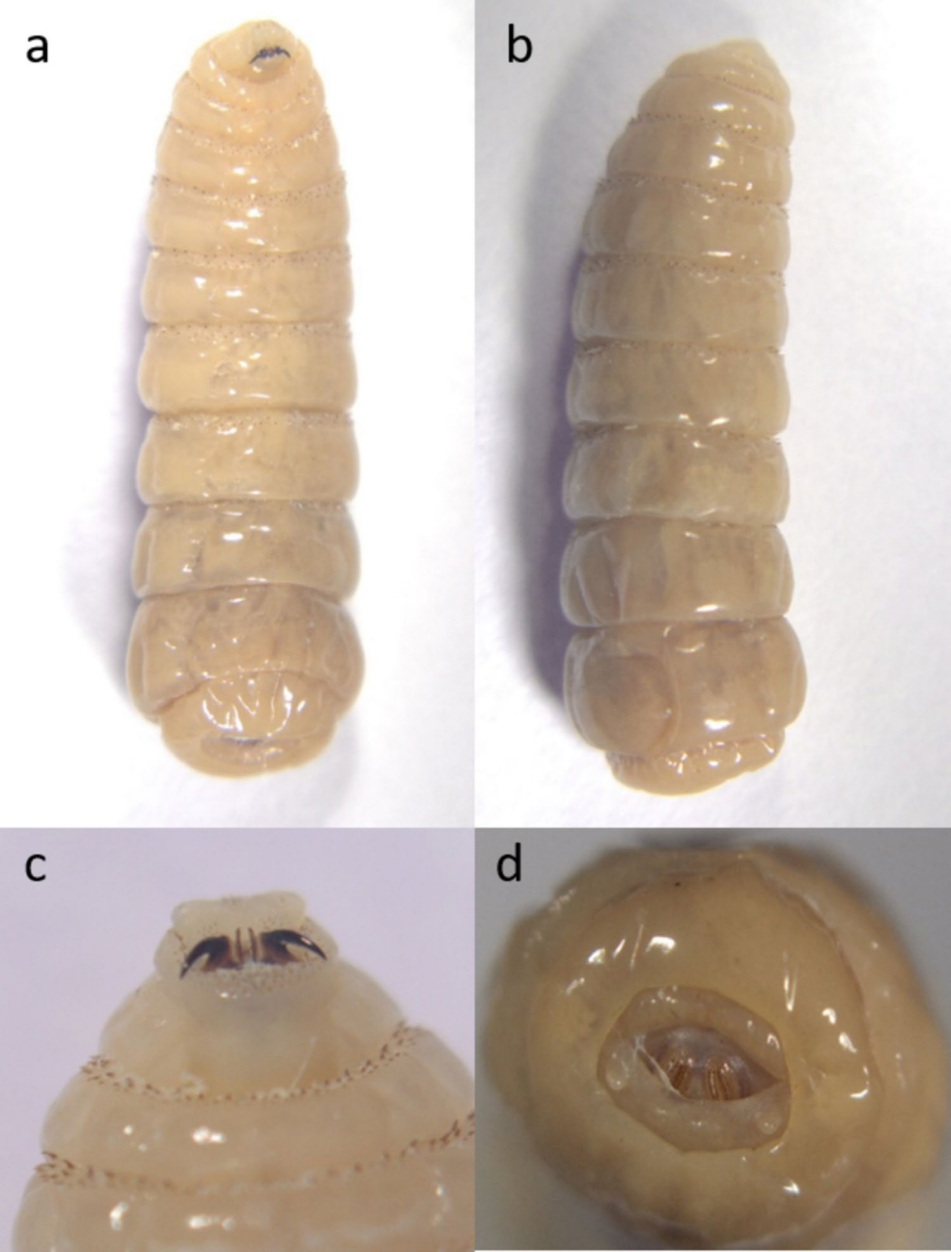
*G. intestinalis* L2 morphology from **a** ventral, **b** dorsal, **c** ventro-cranial, and **d** caudal view

**Fig. 5:**
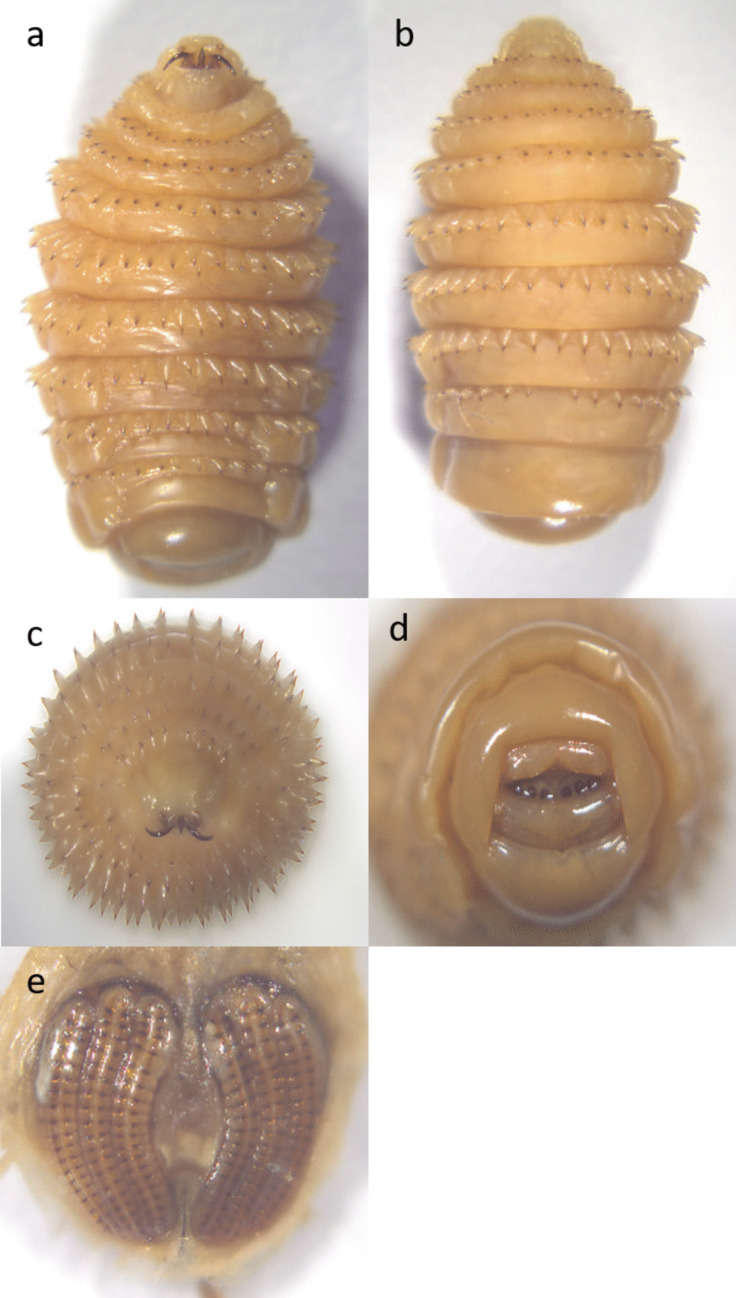
*G. nasalis* L3 morphology from **a** ventral, **b** dorsal, **c** cranial, **d** caudal, and **e** spiracular plates view

**Fig. 6:**
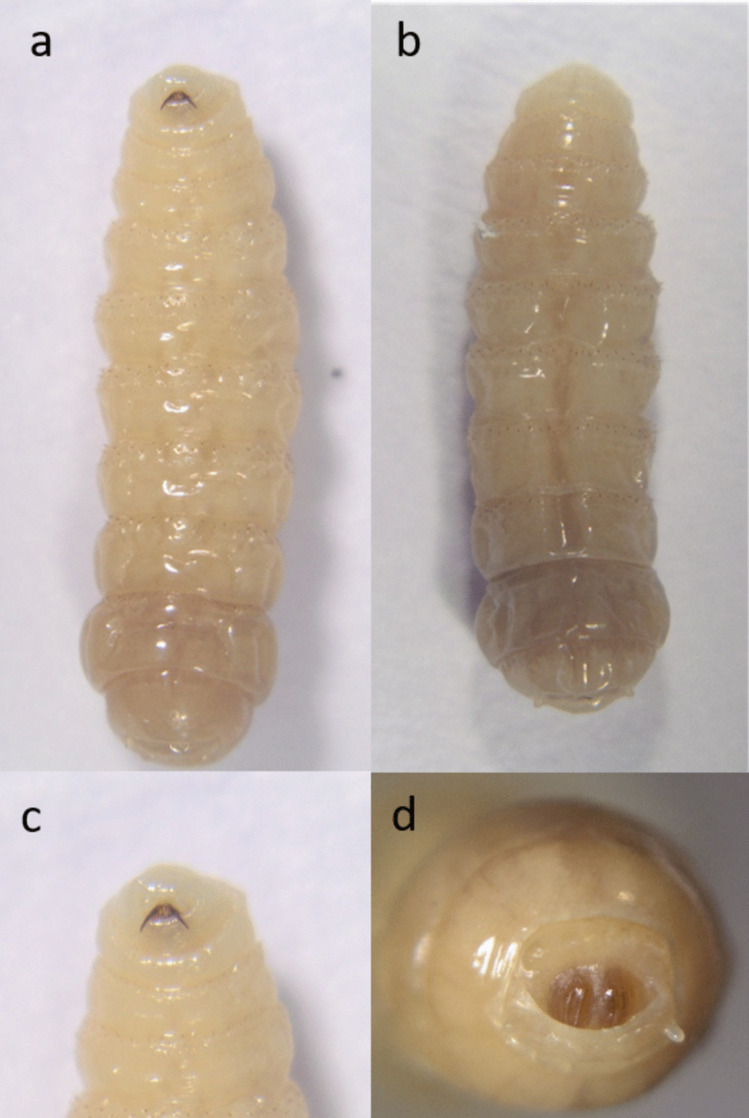
*G. nasalis* L2 morphology from **a** ventral, **b** dorsal, **c** ventro-cranial, and **d** caudal view

Out of the 394 horses, 211 were infected with L2 or L3 stages of *Gasterophilus* spp. (prevalence 53.55%, CI 95% 48.6–58.5%). Two species of *Gasterophilus* were identified: *G. intestinalis* (prevalence 52.0%, CI 95% 47.1–57.0%) (Figs. 3 and 4) and *G. nasalis* (prevalence 14.0%, CI 95% 10.5–17.4%) (Figs. 5 and 6). Coinfection with both species was recorded in 49 horses (prevalence 12.4%). The detailed prevalence by horse categories is shown in Table 1.

**Table 1:** The prevalence (%) across *Gasterophilus* species and larval stages

		Gender		Group age (years)			Month											
		Male	Female	< 3	≤ 3– < 10	≥ 10	Jan	Feb	March	April	May	June	July	Aug	Sept	Oct	Nov	Dec
G. SPP	Total	55.6	51.9	53	50	54.4	57.5	56.9	47.5	60	50	45	60	45	28.6	58.3	75	56.3
	L3	43.8	45.8	48.5	37.5	45.6	57.5	56.9	47.5	60	50	45	60	30	4.8	22.2	50	56.3
	L2	25.3	20.8	18.2	26.8	22.2	27.5	15.7	7.50	0	0	0	0	22.5	23.8	52.8	67.5	50
G. INT	Total	53.9	50.5	53	46.4	52.9	57.5	54.9	47.5	60	50	45	60	40	28.6	52.8	72.5	56.3
	L3	42.1	43.1	47	33.9	43.4	57.5	54.9	47.5	60	50	45	60	30	4.8	8.3	42.5	56.3
	L2	21.3	18.2	20	25	20.6	27.5	15.7	7.5	0	0	0	0	10	23.8	47.2	65	50
G. NAS	Total	14	13.9	16.7	7.1	14.7	12.5	5.9	5	13.3	7.5	22.5	26.7	20	0	30.6	20	0
	L3	10.7	12.5	15.2	5.4	12.1	12.5	5.9	5	13.3	7.5	22.5	26.7	12.5	0	16.7	17.5	0
	L2	6.2	2.3	1.5	1.8	5.1	0	0	0	0	0	0	0	12.5	0	25	5	0

**Fig. 7:**
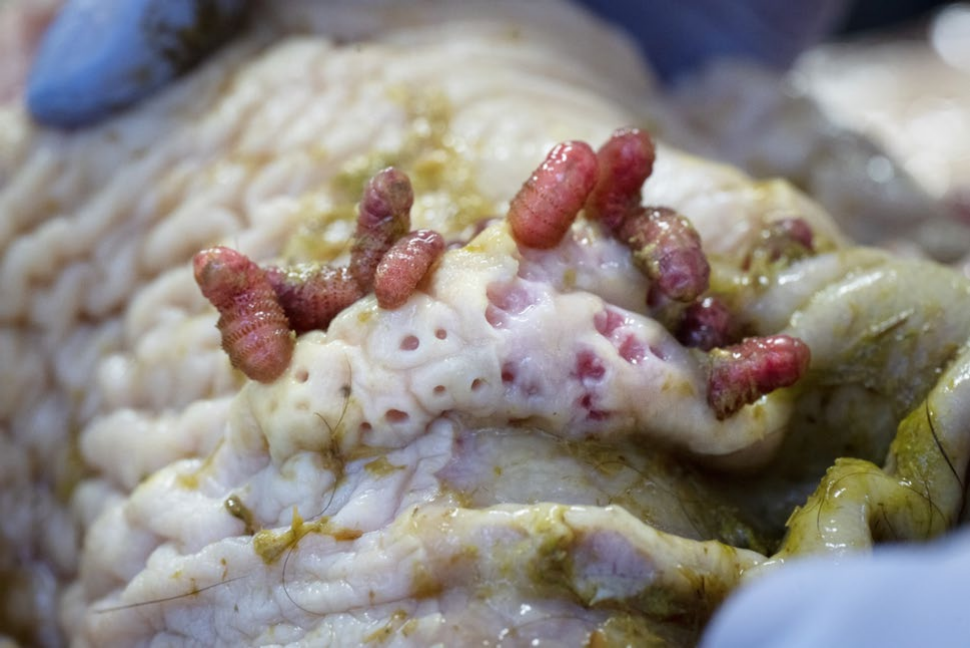
*G. intestinalis* (stomach)

**Fig. 8:**
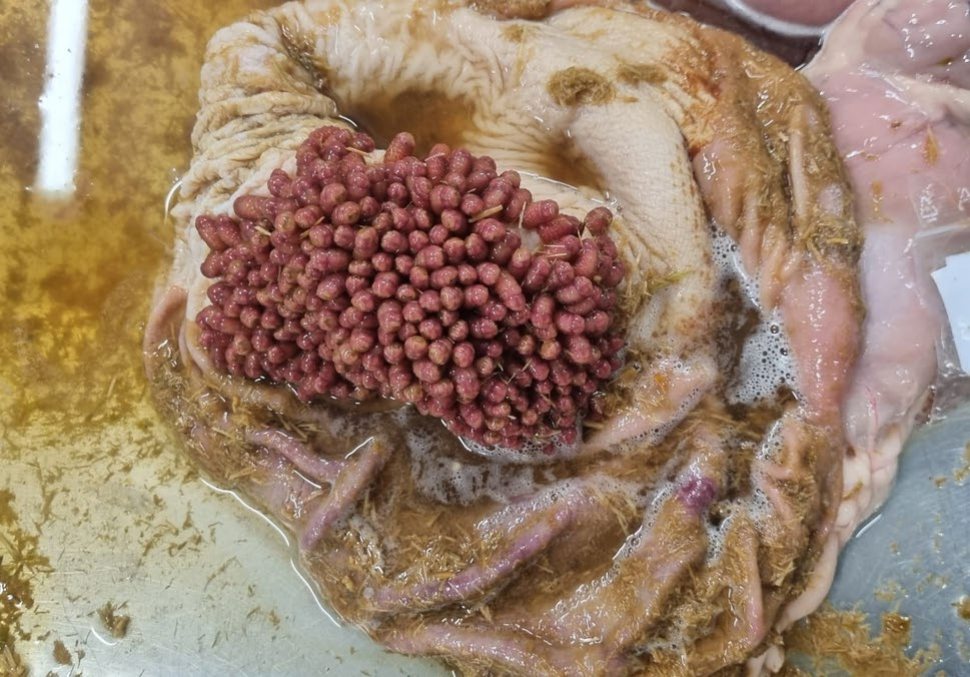
*G. intestinalis* (stomach)

**Fig. 9:**
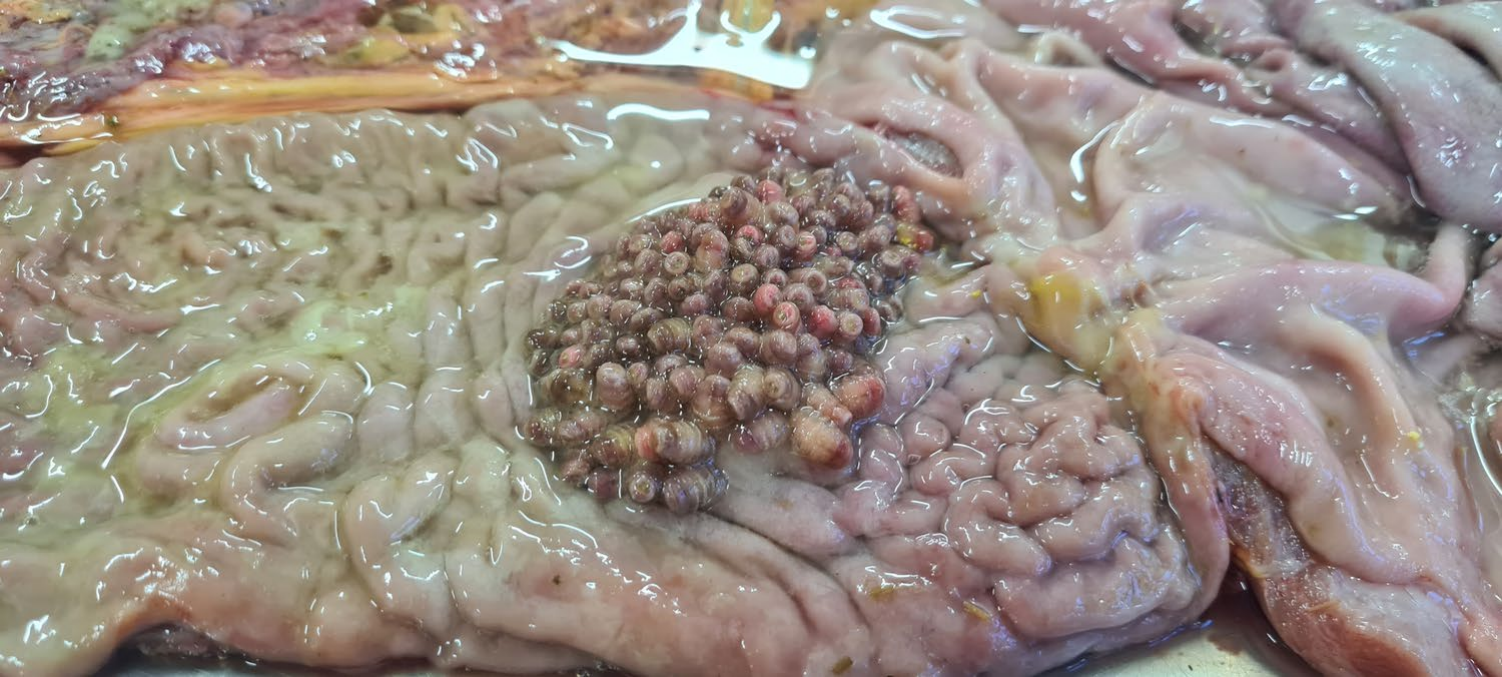
*G. nasalis* (duodenum)

**Fig. 10:**
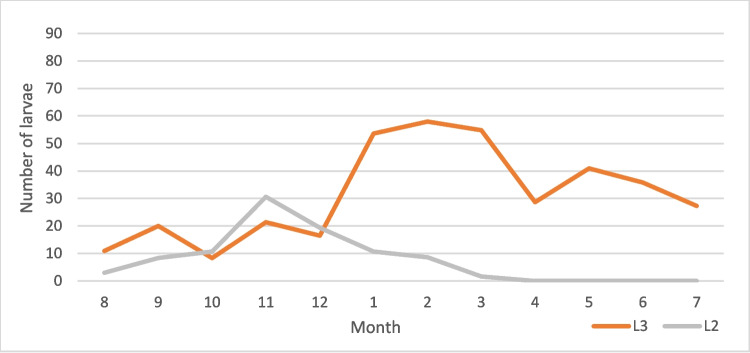
Mean intensity of *G. intestinalis* by month

**Fig. 11:**
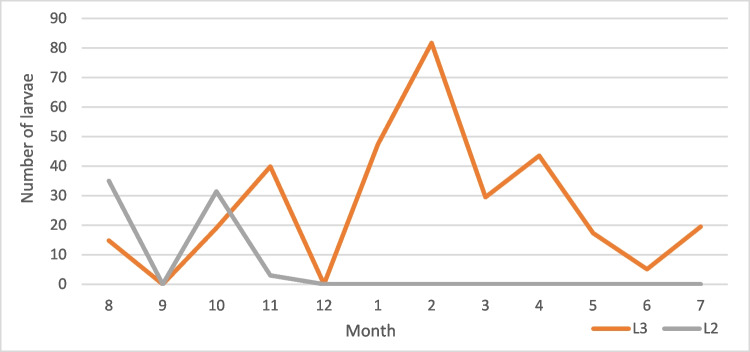
Mean intensity of *G. nasalis* by month

**Fig. 12:**
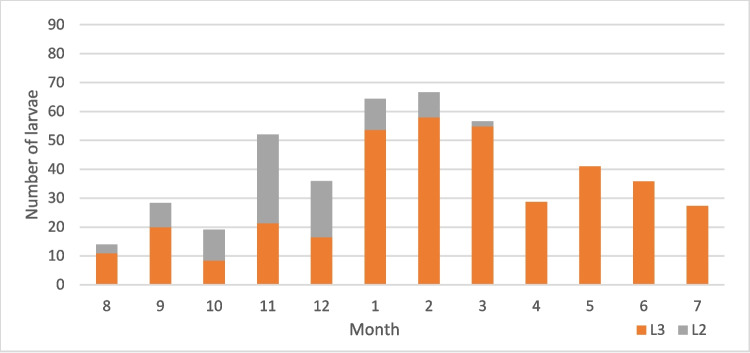
Mean intensity of *G. intestinalis* by month

**Fig. 13:**
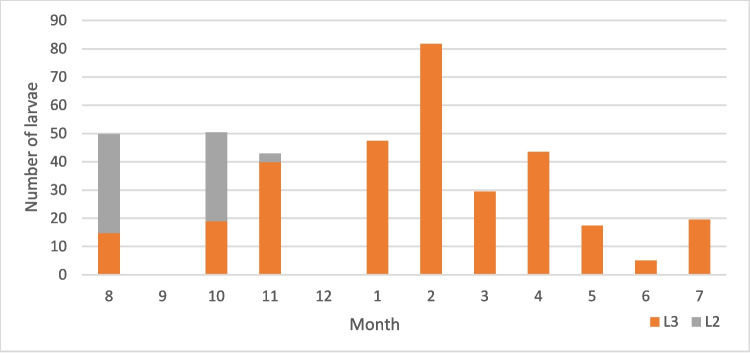
Mean intensity of *G. nasalis* by month

A total of 9759 larvae were collected, of which 7916 were L3 and 1843 were L2. The larvae were collected from the stomach (*G. intestinalis*—Figs. 7 and 8) and duodenum (*G. nasalis*—Fig. 9). Data on intensity, mean intensity, and abundance are shown in Table 2 and Figs. 10, 11, 12, and 13.

**Table 2:** Examined and positive horses and recovered larvae from *Gasterophilus* species across gender, age, and month

	No	*Gasterophilus* spp*.*						*G. intestinalis*						*G. nasalis*					
		M. int		M. ab		Max. int		M. int		M. ab		Max. int		M. int		M. ab		Max. int	
		L3	L2	L3	L2	L3	L2	L3	L2	L3	L2	L3	L2	L3	L2	L3	L2	L3	L2
Male	178	42	15.8	18.4	4	295	103	40.8	22	15.5	2.3	295	138	27	27.3	2.9	3.9	98	103
Female	216	46.9	25.2	21.5	5.3	420	138	36.8	10.8	17.6	4.5	332	46	31.5	32.8	3.9	0.8	139	68
< 3	60	49.9	29.3	24.9	5.9	219	85	40.2	23.7	19.4	4.7	219	85	33	68	5.5	1.1	72	68
≤ 3– < 10	51	26.7	25	9.9	6.9	137	138	28	26.9	9.3	6.8	137	138	11.7	1	0.7	0.02	17	1
≥ 10	283	46.2	17.8	20.9	4	420	103	40.3	13.1	17,4	2.6	332	55	30.2	28.2	3.5	1.4	139	103
January	40	64	10.7	36.8	3	420	46	53.7	10.7	30.9	3	332	46	47.4	0	5.9	0	129	0
February	51	64.4	8.6	36.6	1.4	295	17	58	8.6	31.8	1.4	295	17	81.7	0	4.8	0	139	0
March	40	58	1.7	27.5	0.1	219	2	54.8	1.7	26.1	0.1	219	2	29.5	0	1.5	0	37	0
April	15	38.3	0	23	0	117	0	28.7	0	17.2	0	60	0	43.5	0	5.8	0	57	0
May	40	43.6	0	21.8	0	167	0	41	0	20.5	0	166	0	17.3	0	1.3	0	31	0
June	40	43.4	0	19.6	0	175	0	35.8	0	16.1	0	139	0	15.3	0	3.5	0	39	0
July	15	36	0	21.6	0	85	0	27.3	0	16.4	0	76	0	19.5	0	5.2	0	64	0
August	40	11.4	20.8	5.1	4.7	71	103	10.9	3	3.3	0.3	61	7	14.8	35	1.9	4.4	47	103
September	21	3.3	8.4	1	2	20	22	20	8.4	1	2	20	22	0	0	0	0	0	0
October	36	6.6	24.5	3.9	12.9	55	86	8.3	10.7	0.7	5.1	14	46	19	31.4	3.2	7.9	55	68
November	40	21.4	29.7	16.1	20.7	185	138	21.4	30.6	9.1	19.9	113	138	39.9	3	7	0.2	98	5
December	16	16.4	19.4	9.3	9.7	32	39	16.4	19.4	9.3	9.7	32	39	0	0	0	0	0	0
Total	394	44.7	20.5	20.1	4.7	420	138	39	16.8	16.6	3.5	332	138	29.6	29	3.5	1.2	139	103

The chi-square (*χ*^2^) test did not show any significant differences (*p* < 0.05) between the prevalence (*Gasterophilus* spp., *G. intestinalis*, and *G*. *nasalis*) and month of sampling, gender, age, and colour, except for the monthly prevalence of *G. nasalis* (*χ*^2^ = 28.044, df = 11, *p* = 0.003). The Kruskal–Wallis test showed no correlation between mean intensity and mean abundance across gender, age, and colour. However, there was a strong seasonal variation in mean intensity and mean abundance for both *G. intestinalis* and *G. nasalis* and their coinfections (Table 3).

**Table 3:** Kruskal–Wallis test significant results

Category	Stage	Mean abundance				Mean intensity			
		*χ*^2^	df	*N*	*p*	*χ*^2^	df	*N*	*p*
*Gasterophilus* spp.	Total	23.20	11	394	0.017	22.34	11	211	0.022
	L3	39.32			< 0.001	20.31		177	0.041
	L2	118.54			< 0.001	16.26		90	0.023
*G. intestinalis*	Total	28.30			0.003	43.071		205	< 0.001
	L3	49.95			< 0.001	25.204		168	0.009
	L2	119.26			< 0.001	16.265		82	< 0.001
*G. nasalis*	Total	26.77			0.005	-		-	> 0.05
	L2	58.98			< 0.001	-		-	> 0.05

The post hoc pairwise comparisons using Dunn’s method with a Bonferroni correction for the mean abundance indicated a significant difference (*p* < 0.05) for the monthly mean intensity and mean abundance. The total L2 mean abundance varied significantly between November and the rest of the months except September, October, and December. For L2 of *G. intestinalis*, the month with the most significant difference was also November, with a *p* < 0.001 for all the months except October and December, while for L2 *G. nasalis*, the results indicated that October had the most significant comparisons for all the months except August and December. For L3 of *G. intestinalis* and *Gasterophilus* spp., there was a significant decrease in the mean abundance between January–May and September–October. Regarding the total mean abundance, there were significant differences for *Gasterophilus* spp. and *G. intestinalis* between August and November and September and November. The post hoc comparison for the mean intensity revealed significant differences only for *G. intestinalis*. Significant differences were found between August and October (for L2); August and February and March (for L3); and August and January, February, March, May, and November (all larval stages) (Tables 4, 5, and 6).

**Table 4:** Post hoc pairwise comparisons significant results for *G. intestinalis* - mean intensity

Total	Aug	Nov., Jan., Feb., March, May	*p* < 0.05
	Oct	Nov., Jan., Feb., March	
L3	Aug	Feb., March	
L2	Oct	Nov

**Table 5:** Post hoc pairwise comparisons significant results for *G. intestinalis* - mean abundance

Total	Nov	Aug., Sept	*p* < 0.05
L3	Sept	Jan, Feb, March, May	
	Oct	Jan, Feb, March, May	
L2	Oct	Feb, March, April, May, June, July, Aug	
	Nov	Jan, Feb, March, April, May, June, July, Aug, Sept	
	Dec	March, April, May, June, July, Aug

**Table 6:** Post hoc pairwise comparisons significant results for *Gasterophilus* spp.

Mean abundance—*Gasterophilus* spp.			
Total	Nov	Aug, Sept	*p* < 0.05
L3	Sept	Jan, Feb, March, May	
	Oct	Jan, Feb	
L2	Oct	Feb, March, April, May, June, July	
	Nov	Jan, Feb, March, April, May, June, July, Aug, Sept	
	Dec	March, April, May, June, July	

All 26 amplified specimens were successfully sequenced. For *G. intestinalis*, the BLAST analysis revealed a nucleotide similarity between 99.27 and 100% with other sequences from GenBank (China—MH553411, Canada—MG473228, China—KR230415, China—MH553412). Concerning *G. nasalis*, the results showed a similarity between 94.38 and 99.84% with the sequences from China (MH553408, KR230402, MH553409).

## Discussion

*Gasterophilus* spp. is a widespread botfly with various prevalence across the world. Environmental factors, husbandry conditions, population structure, animal trade and movements, and possibly other factors play a significant role in the diversity and prevalence of *Gasterophilus* spp. (Cope and Catts 1991; Otranto et al. 2005; Pilo et al. 2015; Zhang et al. 2021). In Eurasia, China has the highest diversity of *Gasterophilus* spp. with seven recorded species, with the most prevalent represented by *G. pecorum*, followed by *G. nasalis*, *G. nigricornis*, *G. intestinalis*, *G. haemorrhoidalis*, and *G. inermis* (Liu et al. 2016). High diversity of species in Asia has been reported also in Kazakhstan (G*. intestinalis*, *G. haemorrhoidalis*, *G. nasalis*, *G. pecorum*) (Ibrayev et al. 2015), Iran (*G. inermis*, *G. intestinalis*, *G. nasalis*) (Tavassoli and Bakht 2012), and Turkey (*G. intestinalis*, *G. haemorrhoidalis*, *G. nasalis*) (Özdal et al. 2010). The diversity in other Asian countries, such as South Korea, is lower, with only *G. intestinalis* and *G. nasalis* being reported (Kim 1993), while in Japan, only *G. intestinalis* was found (Yoshihara et al. 1994).

In Europe, the most prevalent species are represented by *G. intestinalis* and *G. nasalis*. According to recent studies, Italy hosts the highest diversity with six recorded species: *G. intestinalis*,* G. nasalis*, *G. haemorrhoidalis*,* G. inermis*,* G. pecorum*, and* G. meridionalis* (Pilo et al. 2015), followed by Russia with five species (*G. intestinalis*, *G. pecorum*, *G. nasalis*, *G. nigricornis*, and *G. haemorrhoidalis*) (Reshetnikov et al. 2014). A lower diversity was reported in France (*G. intestinalis*, *G. nasalis*, *G. haemorrhoidalis*) (Bernard et al. 1994). As in our study, *G. intestinalis* and *G. nasalis* were the only species found in the UK (Edwards 1982; Sweeney 1990), Poland (Sobieszewski 1967; Gawor 1995; Gawor et al. 2006; Kornaś et al. 2006, 2007, 2010; Niedźwiedź et al. 2013), Germany (Rehbein et al. 2013), and Ukraine (Slivinska et al. 2006). *G. intestinalis* was the only identified species in Belgium (Agneessens et al. 1998) and the Netherlands (Borgsteede and van Beek 1998).

In Australia and the Americas, the only recorded species are *G. intestinalis* and *G. nasalis* (Mfitilodze and Hutchinson 1989; Bucknell et al. 1995; Lyons et al. 2000, 2003, 2018; Sequeira et al. 2001; Rodrigues Felix et al. 2007).

Since the last comprehensive study in Romania (more than 73 years ago), the diversity identified in the current study decreased from 5 to 2 species. Several factors could explain this situation. Species such as *G. haemorrhoidalis*, *G. inermis*, or *G. pecorum* are generally reported to have a low prevalence in Europe, so a higher number of samples would be required to detect them. Moreover, the introduction and wide use of macrocyclic lactones can be responsible for a reduction in prevalence, intensity, distribution, and diversity. In addition, also the horse population decreased from 1,100,000 in 1965 to 323,000 in 2022 (Bostan et al. 2020; https://www.Statista.Com/Statistics/1255992/Romania-Number-of-Horses/, 2023). The only country with a common border with Romania, where an epidemiological study was performed recently, is Ukraine with similar epidemiological patterns (Slivinska et al. 2006).

The seasonality patterns of *G. intestinalis* larvae can be resumed at a peak of L3 between January and March and the lower values in October. For L3 of *G. nasalis*, the intensity reached the peak in January–February, and the lowest intensity values were recorded in September. In the case of L2, *G. intestinalis* reached its peak in November, while for *G. nasalis*, the L2 peak was in October. L2 was found between August and February for *G. intestinalis* and between August and December for *G. nasalis*. Hence, we can conclude that although the general seasonal pattern between the two species is similar, there are slight differences. According to life cycle data (Zumpt 1965; Otranto et al. 2005), between the deposition of the eggs and the appearance of L2 in the digestive tract, there are approximately 6 weeks, while the larvae may remain alive inside the egg for weeks or even months in order to the environment temperature. For our case, this suggests that the adult *Gasterophilus* activity in Romania begins in June–July and ends in October–November.

Despite the seemingly shorter activity period for *G. nasalis*, this species reached higher larval intensities than *G. intestinalis*. This is in apparent contradiction with the literature data on the egg batch size for the two species: 400–1000 eggs for *G. intestinalis* and 300–500 eggs for *G. nasalis* (Colwell et al. 2006). This situation could be explained either by a lower survival rate (Pilo et al. 2015) of the larval stages of *G. intestinalis* due to different locations in the host or migration patterns or by the difference in the egg deposition sites. Similarly to the majority of the previous studies, we did not find any significant differences between prevalence and mean intensity across genders and age groups (Pilo et al. 2015).

Our study is the first to assess the influence of horse coat colour on the prevalence and intensity of *Gasterophilus* infestations. The statistical analysis did not show any difference between the values we obtained (prevalence; intensity) and the group colours of the horses included in our study (Horváth et al. 2010). Nevertheless, in order to perform a better assessment of the influence of the colour, there are other factors to take into consideration as the mattness or the brightness.

## Conclusions

Overall, we consider the prevalence of *Gasterophilus* infection to be high in Romania, with a high seasonal pattern which should be considered in the differential diagnosis of gastro-intestinal diseases by the practitioners, as more than half of the horses were affected. Despite the lack of the assessment of clinical signs in our study (due to the employed methodology), we consider equine gasterophilosis to be an important welfare issue in horses in Romania, and probably elsewhere. The implementation of targeted control measures, even without a reliable tool for in vivo diagnosis, should be implemented mainly considering the seasonal patterns. Hence, macrocyclic lactones should be applied in horses in September–October, before the peak larval intensity.

## Supplementary Information

Below is the link to the electronic supplementary material.

Supplementary file1 (TIF 32208 KB)

ESM 1 (PNG 1255 kb)

Supplementary file2 (XLSX 77 KB)

## Data Availability

No datasets were generated or analysed during the current study.

## Abbreviations

PCR: Polymerase chain reaction
DNA: Deoxyribonucleic acid
COI: Cytochrome c oxidase subunit 1
Bp: Base pair
M. ab: Mean abundance
M. int: Mean intensity
