# Supplementary figures and images for: Gasterophilus in horses from Romania: diversity, prevalence, seasonal dynamics, and distribution
Source: Parasitol Res. 2024 Dec 23;123(12):416. doi: 10.1007/s00436-024-08419-3 (PMC11663820; doi:10.1007/s00436-024-08419-3)

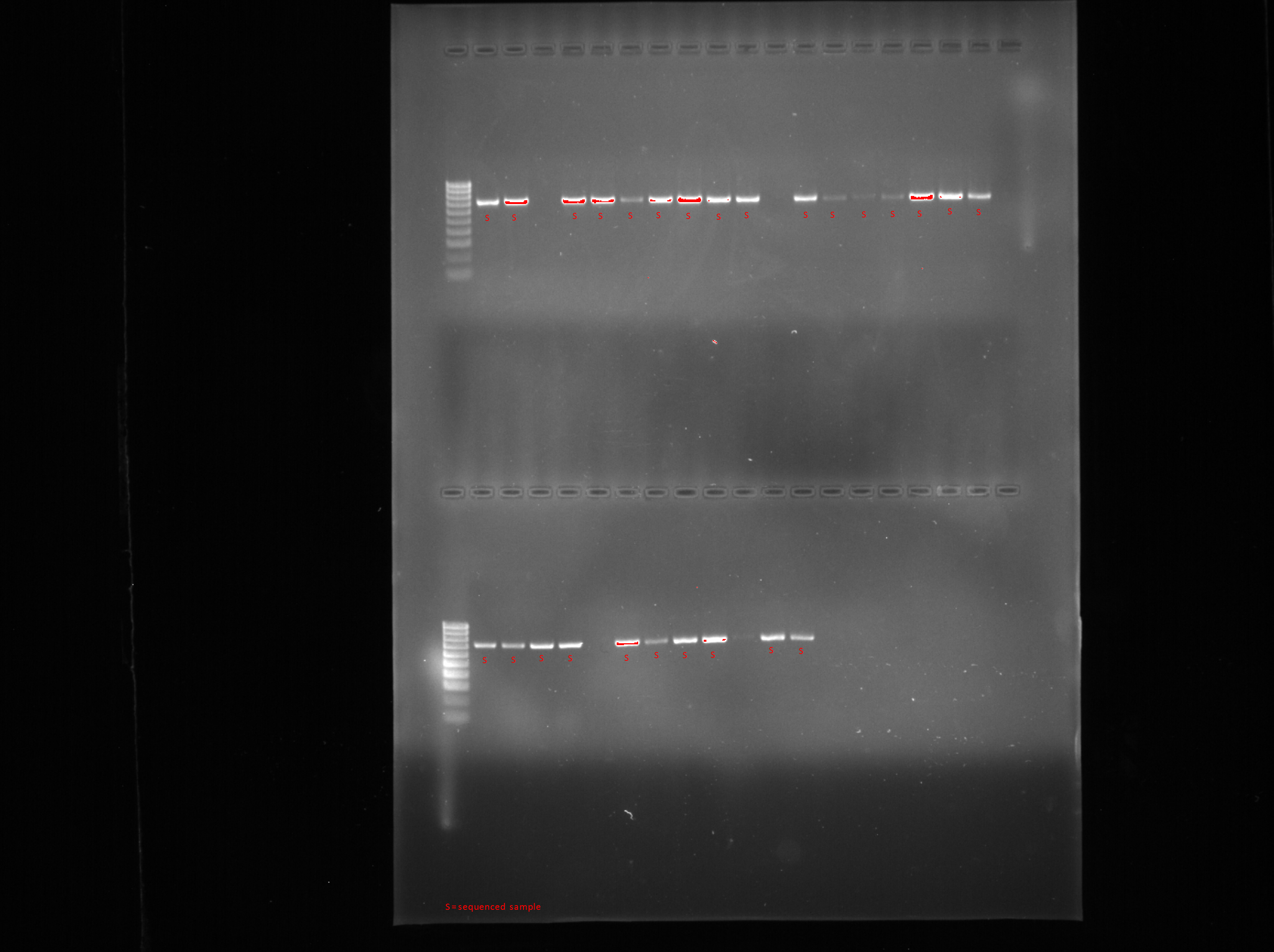

Supplement: Supplementary file 1 — Supplementary file1 (TIF 32208 KB) [file 436_2024_8419_MOESM1_ESM.tif]

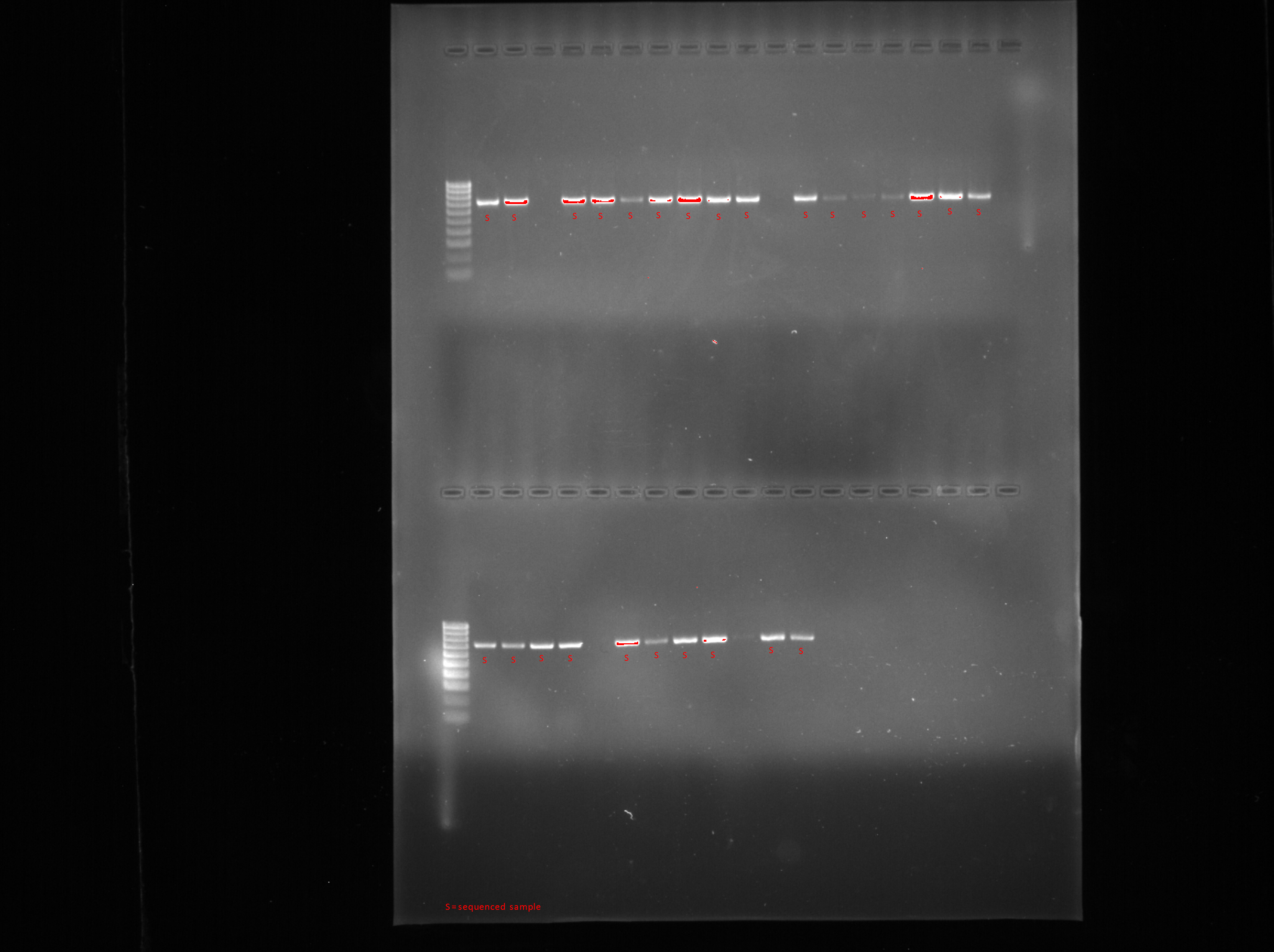

Supplement: Supplementary file 2 — (PNG 1255 kb) [file 436_2024_8419_Fig14_ESM.png]
